# Clinical Outcomes of Arthroscopic Annular Release Around the Greater Trochanter in Severe Gluteal Muscle Contracture Cases: A Technical Note and Case Series

**DOI:** 10.1111/os.70427

**Published:** 2026-09-16

**Authors:** Zi‐Ren Zhou, Ruo‐Fan Li, Fei‐Er Song, Fan Yang, Jian‐Quan Wang, Jia‐Yi Shao, Hong‐Jie Huang

**Affiliations:** ^1^ Department of Sports Medicine Peking University Third Hospital, Institute of Sports Medicine of Peking University Beijing China; ^2^ Beijing Key Laboratory of Sports Injuries Beijing China; ^3^ Engineering Research Center of Sports Trauma Treatment Technology and Devices, Ministry of Education Beijing China

**Keywords:** arthroscopic annular release, clinical outcome, greater trochanter, minimally invasive surgery, severe gluteal muscle contracture

## Abstract

**Objective:**

Severe gluteal muscle contracture often requires surgical release, yet conventional open procedures are associated with extensive soft tissue trauma, significant postoperative pain, and prolonged rehabilitation. This study aimed to introduce an arthroscopic annular release technique for severe gluteal muscle contracture and evaluate its clinical outcomes.

**Methods:**

A retrospective review was conducted using data from patients with severe gluteal muscle contracture who underwent arthroscopic treatment between September 2019 and October 2022. Inclusion criteria were patients presented positive preoperative signs, including squatting and crouching impairment, difficulty in crossing the legs, a positive Ober's sign, with or without clicking sounds during hip rotation. The annular release treatment was done around the greater trochanter, which has a sparse distribution of muscle and neuromuscular tissues. Arthroscopic observation and intraoperative physical examination were used to assess the adequacy of the release. Additionally, clinical assessments included the Gluteal Muscle Contracture Disability Scale (GDS), Visual Analogue Scale (VAS), and pain frequency, which were evaluated pre‐ and postoperatively. A paired Student's t‐test was applied to compare the GDS, VAS, and pain frequency scores before and after surgery.

**Results:**

A total of 22 patients were included in the final analysis, with an average follow‐up of 2 years. All operations were successfully completed, with no intraoperative conversion to alternative surgical methods. After surgery, most patients achieved satisfactory postoperative outcomes; isolated limitations persisted in one patient who reported persistent squatting difficulty, and two patients continued to have trouble crossing their legs. The GDS score was 43.8 ± 13.3 preoperatively and 72.7 ± 9.6 at the final follow‐up, showing a statistically significant improvement (*p* < 0.01). The VAS score was 1.8 ± 2.0 preoperatively and 0.9 ± 1.3 postoperatively, with a significant difference observed (*p* = 0.015). Pain frequency was 2.4 ± 2.7 preoperatively and 0.8 ± 1.2 at final follow‐up, with a statistically significant improvement (*p* < 0.01). No neurologic injuries or other complications were observed during the follow‐up period.

**Conclusion:**

Arthroscopic annular release around the greater trochanter showed adequate contracture band exposure and favorable clinical outcomes in this retrospective case series. It may be a feasible minimally invasive option for severe gluteal muscle contracture.

## Graphical abstract

This study describes a dual‐posterior arthroscopic release technique for severe gluteal muscle contracture that enables complete release around the greater trochanter, direct visualization of the sciatic nerve, and yields significant functional improvement with a low complication rate at a minimum 2‐year follow‐up.

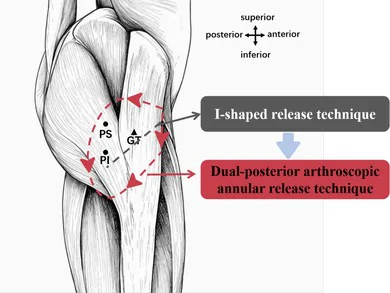

## 1 Introduction

Gluteal muscle contracture (GMC) is mainly characterized by the contracture of the gluteal muscles, iliotibial band (ITB), and associated fascia [1, 2]. Acquired gluteal contracture is the most common subtype of GMC and is generally considered to be closely associated with repeated intramuscular injections to the buttocks during childhood; the younger the age at which injections are administered, the higher the prevalence [3, 4]. The clinical manifestations of GMC typically include hip dysfunction and pelvic deformity. Patients present with a “snapping hip” and exhibit restricted range of motion (ROM) of the hip joint, particularly, in adduction and internal rotation [5, 6, 7].

Surgical intervention is generally considered for symptomatic patients with established GMC who fail conservative treatment [2, 7]. Traditional open surgery provides adequate surgical field exposure, enabling surgeons to resect contracture bands under direct visualization; however, a larger incision entails a greater degree of soft tissue trauma, which can lead to the formation of new contractures [2, 8]. Additional drawbacks of traditional open surgery include extensive surgical injury, cosmetically undesirable scars, hematoma formation, wound‐related complications, and prolonged recovery [6, 7, 9]. In 2009, Liu et al. [10] introduced arthroscopic surgery for GMC. Compared with open surgery, arthroscopic procedures may reduce tissue trauma and intraoperative blood loss and facilitate early postoperative rehabilitation [11].

Although arthroscopic surgery is minimally invasive, in severe cases, challenges such as inadequate exposure of the posterolateral region, nonstandardized portal placement, and reliance on the surgeon's experience for deep contracture release and nerve management persist [10, 12, 13, 14]. This necessitates an expanded release range, which in turn elevates the risk of injury to surrounding muscular, vascular, and neural structures—particularly the sciatic nerve. To address these challenges, we developed a novel dual‐posterior‐portal, full‐quadrant annular release technique around the greater trochanter (GT) for the treatment of severe GMC. This approach enables adequate exposure of contracture bands and facilitates intraoperative identification, visualization, and protection of the sciatic nerve. Additionally, this technique enables adequate exposure of contracture bands (especially the deep gluteal muscle fiber bundles) and allows clear identification of the sciatic nerve's course intraoperatively, facilitating direct visualization and active protection of the sciatic nerve. The C‐shaped release technique is a stepwise, non‐circumferential approach, and its release extent is determined by intraoperative physical examination findings. In contrast, the present annular release technique achieves a full‐quadrant, anatomically oriented release around the GT in a single procedure. Compared with other arthroscopic release techniques, this procedure leverages the anatomical adaptability of the dual posterior approach to achieve full‐quadrant release under direct visualization and active protection of the sciatic nerve, marking a surgical strategy upgrade from experience‐oriented to anatomy‐oriented approach in the treatment of severe GMC.

The purposes of this study were as follows: (1) to describe the technical details of this annular release technique and (2) to investigate the clinical outcomes of this technique. We hypothesized that this technique would improve clinical outcomes while allowing intraoperative visualization and protection of the sciatic nerve.

## 2 Materials and Methods

### 2.1 Patients' Selection

**FIGURE 1:**
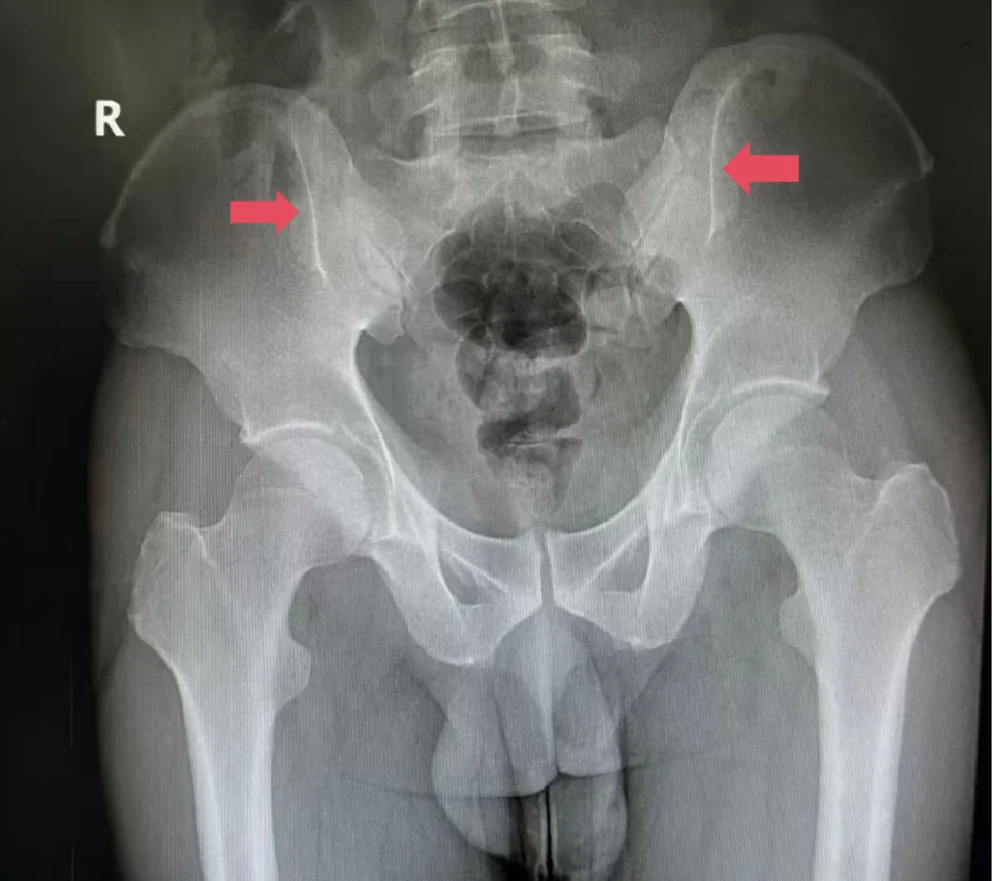
X‐ray showed dense iliac lines (red arrow).

This study retrospectively reviewed consecutive patients who underwent arthroscopic annular release around the GT for GMC between September 2019 and October 2022. This study was reviewed and approved by the Ethics Committee (M2019193), and written informed consent was obtained from all patients. The inclusion criteria were as follows: (i) patients were diagnosed with severe GMC based on medical history, clinical symptoms, and physical examination. The inclusion process involved two steps: First, patients with dense iliac lines on x‐ray were screened [15] (Figure 1). Second, according to the criteria of Yeet al. [16] and Liu et al. [10], severe GMC was confirmed only in patients presenting with all of the following: inability to bring the knees together while squatting, inability to cross or overlap the legs when seated, a strongly positive Ober's sign, and marked contracture band movement with an audible snap during squatting. Only patients meeting both imaging and clinical criteria were included; (ii) Failure to respond to conservative treatment for at least 6 months, with significant impact on daily activities. All patients had received local injections, though the precise number of injections could not be accurately recalled; (iii) patients who underwent arthroscopic annular release. The exclusion criteria were as follows: (i) patients with intra‐articular or extra‐articular hip pathologies, such as hip osteoarthritis, acetabular labral tears, femoroacetabular impingement (FAI) syndrome, internal snapping hip, hip joint infection, and joint ankylosis; (ii) patients with a history of previous ipsilateral hip operation, incomplete preoperative radiographs and medical record.

### 2.2 Surgical Procedure

#### 2.2.1 Patient Positioning and Portal Marking

**FIGURE 2:**
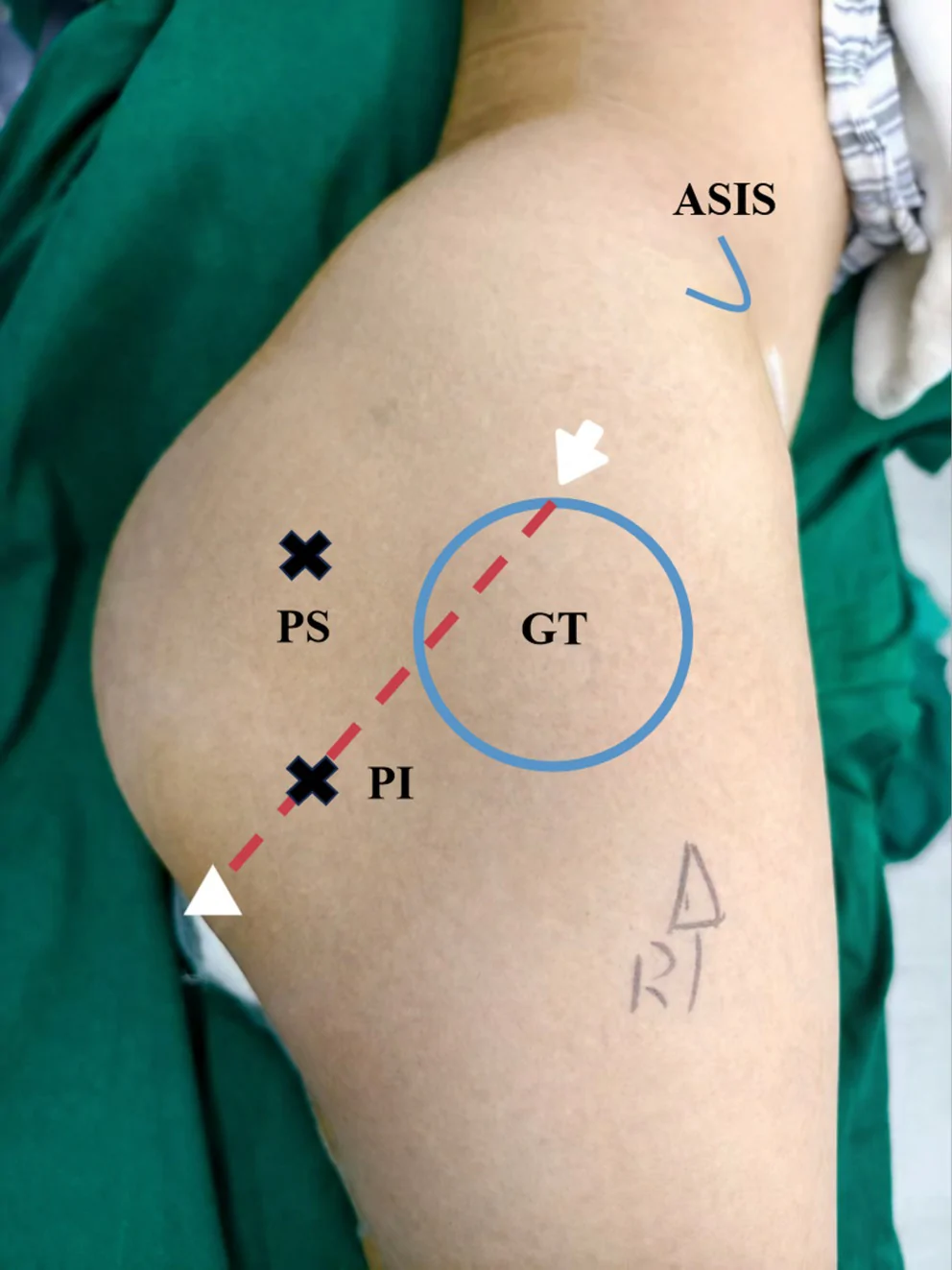
Portals of the surgery. The patient is placed in a lateral decubitus position. The red dashed line indicates the line between the apex of the greater trochanter and the lateral margin of the gluteal fold. The arrow indicates the apex of the greater trochanter, and the triangle indicates the lateral margin of the gluteal fold. ASIS, anterior superior iliac spine. GT, great trochanter; PI, posteroinferior portal; PS, posterosuperior portal.

After anesthesia, the patient was placed in a lateral decubitus position. The outlines of the following structures were delineated: anterior superior iliac spine (ASIS), GT, apex of the GT, and lateral margin of the gluteal fold. Then two portals were marked: the posteroinferior (PI) portal and the posterosuperior (PS) portal. The PI portal was located at the lower one‐third of the line between the lateral margin of the gluteal fold and the apex of the GT. The PS portal was approximately 5 cm superior to the PI portal (Figure 2).

#### 2.2.2 Portal Establishment and Working Space Creation

The arthroscope was introduced through the PS portal, then the operating space above the IT band and gluteal muscle was created by blunt dissection and then fluid inflow. Arthroscopic shaver and radio frequency were introduced to remove the subcutaneous adhesive tissue to allow sufficient exposure of the contracture band. In these severe cases, the contracture band could be located inside the gluteus maximus and gluteus medius muscle, so the key was to remove the adhesive tissue and surrounding adipose tissue above the muscle through a shaver and coagulate the bleeding vessel through radiofrequency meticulously.

#### 2.2.3 Arthroscopic Annular Release

**FIGURE 3:**
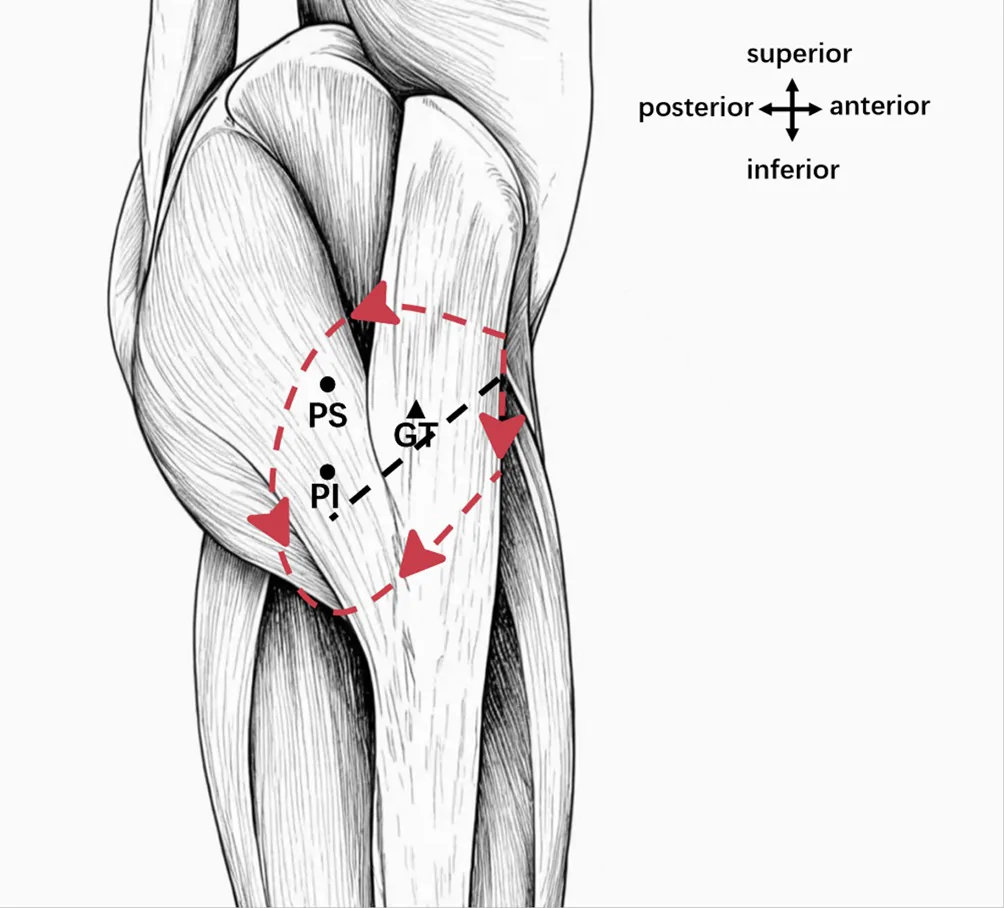
The diagram of the annular release technique. The red dashed line indicates the release range for patients with severe GMC, and the black dashed line indicates the release range for patients with mild GMC. GT, greater trochanter; PI, posteroinferior portal; PS, posterosuperior portal.

**FIGURE 4:**
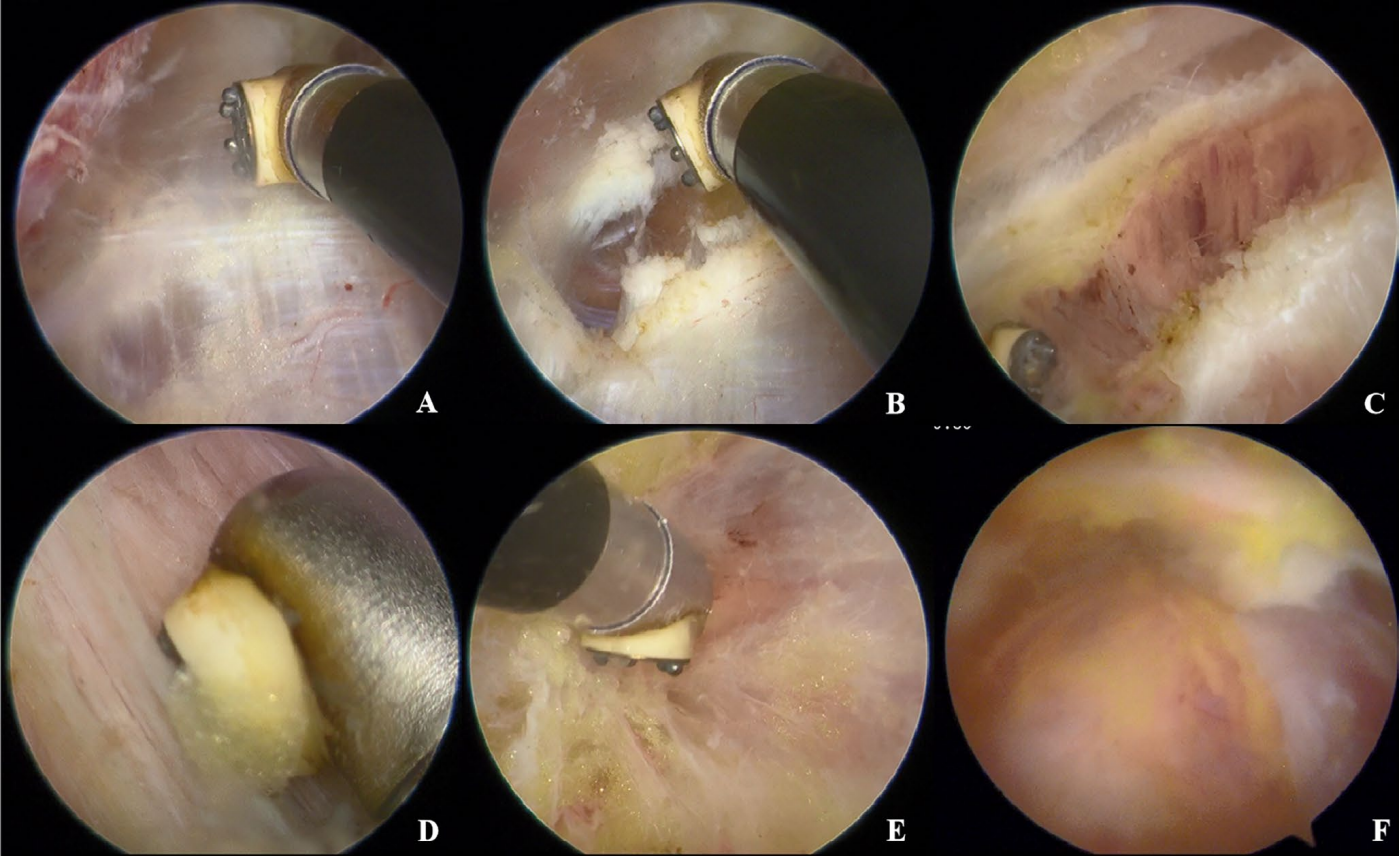
(A) Use a shaver and radiofrequency to expose the surface of the contracture band. (B, C) Release and dissect the exposed contracture band to achieve complete release of the tendinous contracture band. (D, E) Use radiofrequency to release the deep contracture band. (F) Extend the release to normal muscle tissue, separating the contracture band from the underlying structures and restoring hip joint range of motion.

After sufficient exposure, the release procedure began with the use of radiofrequency. First, the contracted IT band was released from anterosuperior to PI above GT, which was identified via internal and external rotation of the hip by the assistant. The upper release extends toward the PS part of the gluteus maximus and then extends downwards to the other end of the release, ultimately forming a fan‐shaped release loop (Figure 3). The key to this procedure lies in accurately identifying the contracture band. Based on our experience, the contracture band appears white, non‐shiny, and inelastic, with no visible normal muscle tissue beneath it; therefore, this serves as the starting boundary for release. After thorough release of these bands, if fibrosis extends into the deeper muscle layers, the release is progressively and meticulously advanced along this boundary toward the deep layers until the deep normal muscle tissue is fully exposed, thereby ensuring complete release of the contracture band. After sufficient release of the contracture band is confirmed, a clinical examination is performed. Complete release is considered achieved when flexion, adduction, and internal rotation are normal, no palpable snapping is detected, and Ober's sign is negative (Figure 4).

#### 2.2.4 Sciatic Nerve Protection and Deep Release

**FIGURE 5:**
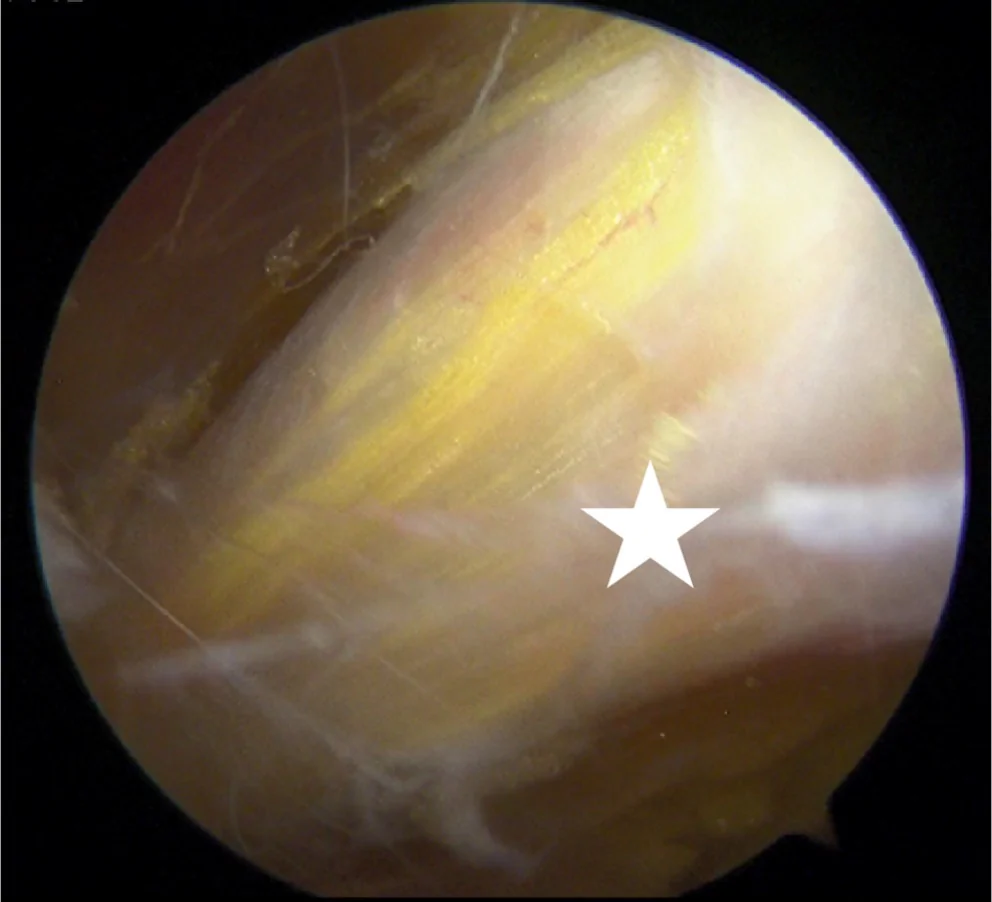
Full exposure of the sciatic nerve (white star).

One major potential risk of this release procedure is iatrogenic injury to the sciatic nerve. In extremely severe cases with full‐thickness contracture of the gluteus maximus, direct exposure of the sciatic nerve may be necessary to ensure safe deep release. In our technique, the contracture bands over the ITB and the superficial layer of the gluteus maximus were first released. During the procedure, the assistant gently touched the toes of the affected limb to detect any sign of sciatic nerve irritation. The natural plane between the anterior aspect of the gluteus maximus and the greater trochanter was then identified and the soft tissue in this plane was bluntly dissected until the sciatic nerve was visualized arthroscopically. After the nerve had been identified, the deeper intramuscular contracture bands within the gluteus maximus were released. This strategy facilitated deep release under direct visualization of the sciatic nerve (Figure 5).

After the release procedure, intraoperative physical examination (passive maximum flexion, adduction, and internal rotation of the hip) was performed to evaluate the hip ROM and whether this was residual contracture.

#### 2.2.5 Technical Challenges and Management Strategies

Several technical difficulties may be encountered. In long‐standing cases with extensive adhesions involving the gluteus minimus or hip capsule, complete release can be technically challenging; we recommend advancing the release along the identified fibrotic boundary while repeatedly assessing hip ROM to verify adequate decompression. When severe adhesions obscure normal tissue planes, slow blunt dissection with meticulous radiofrequency hemostasis helped maintain a clear surgical field. Posterior portal placement is intended to facilitate access to the posterolateral gluteal region. Direct arthroscopic visualization of the nerve in extremely severe cases and continuous monitoring of toe movement are recommended during deep release.

### 2.3 Postoperative Rehabilitation

All patients followed a standardized postoperative rehabilitation protocol, which initiated with isometric muscle contraction exercises immediately after anesthesia recovery. On the second postoperative day, partial weight bearing was authorized, and functional exercises such as muscle strengthening and ROM training began. From the 3rd to the 4th postoperative week, patients gradually resumed normal daily activities and sports engagement.

### 2.4 Clinical Outcomes

Several parameters were used to assess the outcomes of this technique in the present study, including operative time (per hip), the Gluteal Muscle Contracture Disability Scale (GDS), the Visual Analogue Scale (VAS), pain frequency, and complication incidence (recorded per patient). The GDS score was employed to evaluate patients' functional impairment and recovery. All patients were assessed preoperatively and at the final follow‐up. The GDS comprises 13 items: gait, leg‐crossing ability, squatting ability, hip clicking, stair climbing, hip fatigue, hip pain, hip friction, ability to bring knees together, ability to touch ankles while supine, running limitation, standing long jump performance, and hurdling limitation. Based on previous literature and the most commonly reported residual symptoms in GMC patients, three GDS subitems (snapping hip, squatting with knees together, and leg‐crossing) were prespecified as the primary functional outcomes for detailed analysis [17, 18]. The VAS and pain frequency were used to evaluate pain intensity and frequency, respectively.

### 2.5 Follow‐Up Protocol

Patients were followed up at 3, 6, 12, and 24 months postoperatively, with the final follow‐up conducted at the latest time point available for each patient. At each visit, clinical outcomes were assessed through face‐to‐face interviews and physical examinations. The GDS questionnaire was administered by an independent evaluator who was not involved in the surgical procedures. Pain intensity and frequency were recorded based on patient self‐reports. Postoperative complications were documented at each follow‐up visit. For patients unable to attend in‐person visits, telephone interviews were conducted to obtain functional and pain‐related outcome data.

### 2.6 Statistical Methods

All statistical analyses were performed using IBM SPSS Statistics 27 (IBM Corp., Armonk, NY, USA). The Shapiro–Wilk test was used to assess normality. GDS total scores, pain frequency, and VAS scores were normally distributed and are presented as mean ± SD; paired‐sample *t*‐tests were used for preoperative‐to‐postoperative comparisons. GDS subitem scores were non‐normally distributed and are presented as median (IQR); the Wilcoxon signed‐rank test was used for comparisons. *p* Value < 0.05 was considered statistically significant.

The unit of analysis was defined according to the nature of each variable. In this study, all patients with bilateral GMC underwent simultaneous bilateral procedures under the same anesthetic session. Operative time was recorded per hip, as the duration for each side could be documented independently during the sequential procedure. Intraoperative blood loss was recorded per procedure: for bilateral simultaneous surgery, the total blood loss for the entire procedure was documented; for unilateral surgery, the actual blood loss for the affected side was recorded. Physical examination findings (Ober's sign), complications, and GDS scores were recorded per patient, as these variables reflect patient‐level rather than hip‐specific outcomes.

## 3 Results

### 3.1 Characteristics of the Patients

In the annular release group, there were 7 male and 15 female patients, with a mean age of 31.1 ± 7.1 years and a mean body mass index (BMI) of 21.8 ± 3.1 kg/m^2^. All surgeries were performed successfully without intraoperative modifications to the original surgical plan, with a mean postoperative follow‐up of 2 years (Table 1).

**TABLE 1:** Study population: Patient characteristics.

Study population	
Age, mean ± SD, years	31.1 ± 7.1
Sex, *n* (%)	
Women	15 (68.18)
Men	7 (31.82)
BMI, kg/m^2^, mean ± SD	21.8 ± 3.1
Operative side, *n* (%)	
Bilateral	15 (68.18)
Right	5 (22.73)
Left	2 (9.09)
Operative time per hip, mean ± SD, min	49.05 ± 7.18
Intraoperative blood loss,^a^ M (P25, P75), mL	30 (15, 35)

Abbreviations: BMI, body mass index; *N*, number of patients; SD, standard deviation.

^a^ Intraoperative blood loss was recorded per procedure.

### 3.2 Intraoperative Results

Intraoperatively, all 22 patients were found to have dense fibrotic contracture bands involving the ITB and the superficial layer of the gluteus maximus, with extension into the gluteus medius in 8 patients (36.4%). Extensive adhesions obscuring the normal tissue planes were encountered in 6 patients (27.3%), requiring prolonged blunt dissection and meticulous hemostasis. Direct arthroscopic visualization of the sciatic nerve was necessary in 3 patients (13.6%) with full‐thickness gluteus maximus contracture to ensure safe deep release. No major intraoperative vascular injury or nerve transection occurred.

### 3.3 Clinical Outcomes

The preoperative and postoperative scores of the GDS, VAS, and pain frequency are presented in Table 2. The mean preoperative GDS score was 43.8 ± 13.3, which significantly increased to 72.7 ± 9.6 at the final follow‐up (*p* < 0.01). The preoperative VAS score was 1.77 ± 2.07, and it decreased to 0.86 ± 1.25 at the final follow‐up, with a statistically significant difference (*p* = 0.015). Regarding pain frequency, the preoperative value was 2.36 ± 2.72, and it reduced to 0.77 ± 1.15 at the final follow‐up, also showing a significant difference (*p* < 0.01).

**TABLE 2:** Changes of observation indices after arthroscopic annular release around greater trochanter (mean ± standard deviation).

Parameters	Preoperation	Final follow‐up	*t*	*p*
GDS score	43.8 ± 13.3	72.7 ± 9.6^a^	8.772	*p* < 0.01
VAS score	1.77 ± 2.07	0.86 ± 1.25^a^	2.664	*p* = 0.015
Pain frequency	2.36 ± 2.72	0.77 ± 1.15^a^	3.840	*p* < 0.01

^a^ Compare to the preoperative, *p* < 0.05. Data at the final follow‐up (mean follow‐up, 24 months).

No neurovascular injuries occurred intraoperatively in this cohort, and all patients exhibited normal sensory and motor function in both lower extremities postoperatively. At the final follow‐up, Ober's sign was negative in all 22 patients, and no snapping hip was observed in the 37 affected hips. One patient had residual squatting difficulty, and two had difficulty crossing their legs. Significant improvements were observed in the three GDS subitems corresponding to the most common residual symptoms: snapping hip improved from 7.0 (5.5, 7.0) to 10.0 (7.0, 10.0) (*p* = 0.01); squatting with knees together improved from 4.0 (1.0, 4.0) to 10.0 (9.25, 10.0) (*p* < 0.001); and leg‐crossing improved from 0.0 (0.0, 10.0) to 10.0 (10.0, 10.0) (*p* < 0.001). The remaining patients had no squatting or leg‐crossing difficulties. Most patients achieved favorable functional outcomes, and no patient required further surgery.

### 3.4 Surgical Complications

No muscle weakness, surgical hematoma, or neurovascular injury was recorded at the final follow‐up. All patients had good wound healing.

### 3.5 Representative Case Presentation

**FIGURE 6:**
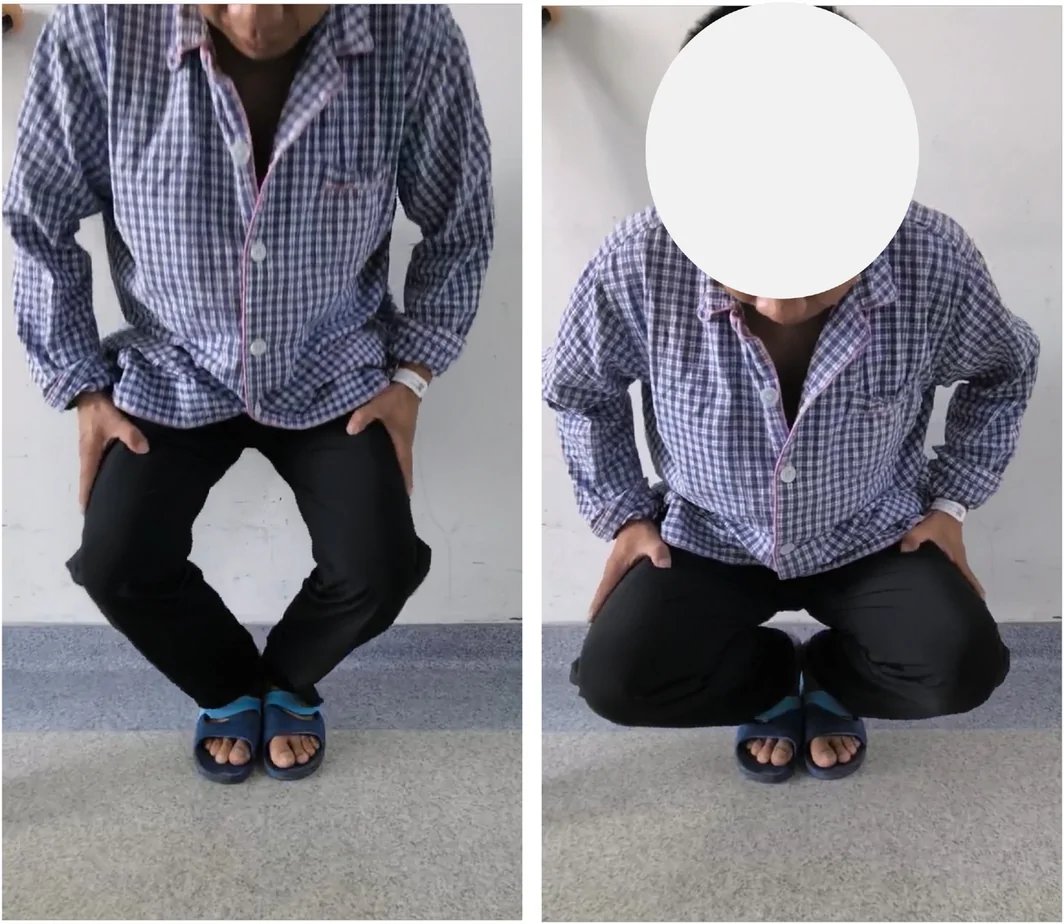
Preoperative functional photograph showing squatting with knees together. The patient presented with marked squatting limitation preoperatively, characterized by severe limitation in squatting with knees together.

To intuitively illustrate the functional recovery achieved with this procedure, a representative patient from the cohort was selected for detailed case description. This patient presented with pronounced squatting limitation preoperatively, characterized by marked limitation in squatting with knees together, as shown in Figure 6.

At the 3‐month postoperative follow‐up, the VAS score decreased from 3 preoperatively to 1, while the GDS score improved from 43 to 56, and Ober's sign converted from positive to negative. By 6 months postoperatively, the VAS score had further declined to 0, with the GDS score increasing to 65; the patient had regained full independence in activities of daily living. At the 1‐ and 2‐year follow‐ups, the GDS score continued to rise to 80, physical examination remained unremarkable, and no postoperative complications were documented.

The functional recovery trajectory of this representative case was consistent with the overall findings of the entire cohort (Table 2), further supporting the efficacy of this procedure in improving hip function.

## 4 Discussion

### 4.1 The Annular Release Technique: Full‐Quadrant Release With Sciatic Nerve Visualization

In this study, dual posterior approaches (PS and PI portals) were used to perform arthroscopic release in patients with severe GMC. These approaches allow the release range to extend around the GT, enabling complete release of the ITB and contracted gluteal fascia. Meanwhile, the surgeon can access the gluteal intermuscular region under direct arthroscopic visualization to release the contracture bands. No nerve injuries were observed in this series. Postoperative follow‐up showed improvements in GDS, pain frequency, and VAS scores.

### 4.2 A Comparative Review of Techniques and Outcomes

GMC is a clinical syndrome characterized by fibrotic contracture and deformation of the gluteal muscles and fascia, caused by multiple factors. This condition leads to hip joint dysfunction and presents with specific gait abnormalities and hip joint physical signs. Treatment options for GMC include conservative treatment and surgical intervention. Generally, nonsurgical treatment is only suitable for patients with mild symptoms, which include massage, physical therapy, short‐wave diathermy, and active‐passive hip joint extension exercises [19] Nonsurgical treatment is more effective in children than in teenagers; however, once contracture is established, nonsurgical treatment typically yields poor outcomes [16]. Therefore, surgical intervention is generally considered for symptomatic patients with established GMC who fail conservative treatment [20, 21].

Previous studies have reported several surgical approaches for GMC, including conventional open surgery and minimally invasive arthroscopic surgery [2, 22, 23, 24]. Conventional open release for GMC has a long history and is associated with advantages such as clear surgical field exposure, reliable release, favorable outcomes, and a low recurrence rate [9, 25]. However, compared to open surgery, the arthroscopic technique offers advantages such as smaller incisions, faster postoperative functional recovery, and fewer complications. It has now been widely used in the treatment of GMC [2, 26, 27]. There are various arthroscopic release techniques have been reported in the literature. Liu et al. [10] reported using inferior, anterosuperior, and PS portals for arthroscopic gluteal contracture release in patients with mild‐to‐moderate lesions. For patients with severe GMC, however, an auxiliary small incision is still required to achieve adequate release. To further improve the surgical treatment of GMC, Tang et al. [12] introduced an arthroscopic C‐shaped release technique around the GT. This technique not only shortens the operative time but also reduces the technical difficulty, and it can be extended to the treatment of severe cases. Gao et al. [13] employed a Type I arthroscopic release using a combined anterior–posterior approach. The results showed that this minimally invasive technique had a high success rate and allowed patients to recover quickly after surgery. Although the above techniques have extended arthroscopy to selected severe GMC cases and reduced open incision reliance, their limitations remain evident compared to annular release: non‐anatomical portal placement, arc‐shaped release with posterolateral blind spots, poor access to deep contracture bands, and nerve avoidance reliant on surgeon experience. To address these issues, we developed a dual posterior approach (PS and PI portals). Its key advantages stem from anatomically optimized portal placement aligned with the posterolateral gluteal region, enabling full‐quadrant release around the GT for complete division of the ITB and contracted fascia. This approach also allows direct arthroscopic access to the gluteal intermuscular space for precise deep release, while providing clear visualization of the sciatic nerve for intraoperative protection. Compared with the isolated anterior superior approach, the dual posterior approach offers superior anatomical adaptability: the anterior superior approach has blind spots in the posterolateral region and cannot fully visualize the sciatic nerve, whereas the dual posterior approach simultaneously covers both the anterior and posterolateral regions, achieving full‐quadrant release with direct nerve protection. These features make the dual posterior approach an effective, reproducible, and minimally invasive option for severe GMC.

### 4.3 Advantages and Disadvantages of the Annular Release Technique

The primary innovation of this study is the use of a dual‐posterior‐approach technique to conduct arthroscopic annular release in patients with severe GMC. This technique offers the following advantages: (i) After inserting the arthroscope through the PS portal, blunt dissection and fluid infusion are employed to create a working space between the ITB and the superficial gluteal muscles. A shaver and radiofrequency are then used to remove subcutaneous adhesions, ensuring proper exposure and relaxation of the contracture bands. (ii) For deep contracture tissues beneath the gluteus maximus and medius, this method allows meticulous removal of adhesions and coagulation of bleeding points, while facilitating visualization of adjacent neurovascular structures.

Although the dual posterior approach offers clear advantages, it has limitations. In severe, long‐standing cases with extensive adhesions involving the gluteus minimus or hip capsule, complete arthroscopic annular release may be technically difficult. In this study, three patients (13.6%) had residual mild functional impairment postoperatively. Possible causes include difficulty in identifying deep boundaries due to dense chronic adhesions, relatively conservative release near neurovascular structures, and differences in postoperative rehabilitation. Additionally, approach‐related complications such as instrument crowding, fluid extravasation, or injury to inferior gluteal nerve branches cannot be entirely avoided. These limitations indicate that although annular release represents a notable advance in minimally invasive treatment for severe GMC, optimal outcomes and low complication rates still depend on rigorous patient selection and surgical experience. Furthermore, as the present study excluded patients with a history of ipsilateral hip surgery, the potential applicability of this technique to revision surgery requires further investigation in dedicated cohorts.

## 5 Limitations

This study has several limitations: (1) The low incidence of severe GMC resulted in a small sample size and a relatively short follow‐up period in this study. Future studies should enroll larger patient cohorts and conduct longer follow‐up to verify these findings. (2) The patient cohort included in this study was relatively young, and the applicability of this surgical technique to elderly populations or broader patient groups remains to be validated. Future studies should expand the age range and demographic diversity of the sample to further assess its generalizability. (3) Our outcome assessment primarily relied on joint mobility and patient satisfaction, without objective radiological criteria. Future studies should incorporate objective tools, such as musculoskeletal ultrasound or MRI to visualize contracture release, and dynamic electromyography to assess gluteal muscle function, to improve validity and reproducibility. (4) This single‐center retrospective study lacks a control group. Future prospective comparative studies are needed to investigate the outcomes between different surgical techniques in severe GMC cases. (5) Although this procedure facilitates sufficient visualization of deep muscular layers and protection of neurovascular structures, it may be associated with increased technical demands, operative time, and intraoperative bleeding, which should be evaluated in future comparative studies. This issue necessitates future improvement via technological modifications in clinical application.

### 5.1 Prospects of Clinical Application

Arthroscopic annular release around the GT showed appropriate contracture band exposure and favorable clinical outcomes in this retrospective case series. It may be a feasible minimally invasive option for severe GMC. These features make it a promising minimally invasive option for severe GMC. However, in long‐standing cases with extensive adhesions, complete release may be technically demanding, and residual contracture bands or approach‐related complications cannot be fully eliminated. Future work should include multicenter prospective studies with larger sample sizes and longer follow‐up to further evaluate long‐term efficacy and safety. With continued refinement, this technique may become a reliable and reproducible minimally invasive treatment for severe GMC.

## 6 Conclusion

Arthroscopic annular release around the GT for severe GMC results in appropriate contracture band exposure and positive clinical outcomes. It may be a feasible minimally invasive option for severe GMC.

## Author Contributions

**Zi‐Ren Zhou:** writing – original draft. **Ruo‐Fan Li:** data curation, software. **Fei‐Er Song:** data curation, investigation. **Fan Yang:** writing – review and editing. **Jian‐Quan Wang:** writing – review and editing, validation. **Jia‐Yi Shao:** writing – original draft, writing – review and editing, data curation, funding acquisition. **Hong‐Jie Huang:** conceptualization, methodology, resources, supervision.

## Funding

Hong‐Jie Huang was supported by the Beijing Natural Science Foundation (L2602041) and the National Natural Science Foundation of China (82372418). Jia‐Yi Shao was supported by the Clinical Medicine Plus X‐Young Scholars Project, Peking University (PKU2026PKULCXQ033) and the Key Clinical Projects of Peking University Third Hospital (BYSYZD2024055).

## Disclosure

The authors' names listed on the full title page are identical to those declared in the ScholarOne submission system. The article title on the full title page is identical to the one declared in the ScholarOne submission system.

## Ethics Statement

Approval for the study was granted through the Peking University Third Hospital review board IRB (Number:M2019056). All patients provided informed consent to participate in this study.

## Conflicts of Interest

The authors declare no conflicts of interest.

## Data Availability

The data that support the findings of this study are available on request from the corresponding author. The data are not publicly available due to privacy or ethical restrictions.

## References

[1] Y. S. Hang , “Contracture of the Hip Secondary to Fibrosis of the Gluteus Maximus Muscle,” Journal of Bone and Joint Surgery. American Volume 61, no. 1 (1979): 52–55. 759436

[2] Y. Jiang , T. Li , L. Wang , et al., “Comparison of Open Surgery Versus Endoscopic‐Assisted Release for Gluteal Muscle Contracture: A Systematic Review and Meta‐Analysis,” Journal of Orthopaedic Surgery and Research 19, no. 1 (2024): 39. 38183149 10.1186/s13018-023-04452-7 PMC10770938

[3] P. S. Bergeson , S. A. Singer , and A. M. Kaplan , “Intramuscular Injections in Children,” Pediatrics 70, no. 6 (1982): 944–948. 6755373

[4] D. V. J. Fernandez and D. M. R. Esteve , “Fibrosis of the Gluteus Maximus: A Cause of Limited Flexion and Adduction of the Hip in Children,” Clinical Orthopaedics and Related Research 156 (1981): 67–78. 7226666

[5] C. G. Brignall , R. M. Brown , and G. D. Stainsby , “Fibrosis of the Gluteus Maximus as a Cause of Snapping Hip. A Case Report,” Journal of Bone and Joint Surgery. American Volume 75, no. 6 (1993): 909–910. 8314831 10.2106/00004623-199306000-00012

[6] S. Rai , C. Meng , X. Wang , et al., “Gluteal Muscle Contracture: Diagnosis and Management Options,” SICOT Journal 3 (2017): 1. 10.1051/sicotj/2016036 PMC5217396 28059055

[7] K. Alves , J. N. Katz , and C. S. Sabatini , “Gluteal Fibrosis and Its Surgical Treatment,” Journal of Bone and Joint Surgery. American Volume 101, no. 4 (2019): 361–368. 30801376 10.2106/JBJS.17.01670 PMC6738551

[8] K. Zha , G. Liu , S. Yang , and F. Cao , “Z‐Plasty for Severe Gluteal Muscle Contracture in Children,” Journal of Orthopaedic Surgery (Hong Kong) 24, no. 3 (2016): 383–386. 28031512 10.1177/1602400323

[9] Y. Ma , J. Gao , F. Zheng , et al., “Gluteus Medius Contracture: A Case Report and Review of the Literature,” Medicine 104, no. 4 (2025): e41311. 39854763 10.1097/MD.0000000000041311 PMC11771728

[10] Y. J. Liu , Y. Wang , J. Xue , et al., “Arthroscopic Gluteal Muscle Contracture Release With Radiofrequency Energy,” Clinical Orthopaedics and Related Research 467, no. 3 (2009): 799–804. 18975040 10.1007/s11999-008-0595-7 PMC2635461

[11] S. Rai , S. Jin , C. Meng , et al., “Arthroscopic Release Using F and C Method Versus Conventional Open Release Method in the Treatment of Gluteal Muscle Contracture: A Comparative Study,” BMC Musculoskeletal Disorders 18, no. 1 (2017): 113. 28302115 10.1186/s12891-017-1484-6 PMC5356281

[12] X. Tang , W. Qi , Y. Liu , et al., “Arthroscopic C‐Shaped Release Around the Greater Trochanter for Gluteal Muscle Contracture,” Orthopaedic Surgery 13, no. 6 (2021): 1765–1772. 34351059 10.1111/os.13103 PMC8523753

[13] S. G. Gao , W. J. Liu , M. Yang , et al., “Clinical Results of Arthroscopic Tight Fibrous Band Release for Adult Moderate‐To‐Severe Gluteal Fibrosis Using Anterior and Posterior Portals: A Retrospective Analysis of 118 Consecutive Cases,” BMC Musculoskeletal Disorders 22, no. 1 (2021): 28. 33407335 10.1186/s12891-020-03885-z PMC7786509

[14] Y. Wu , Y. Xiong , J. Meng , et al., “Arthroscopic I‐Shaped Release Below the Greater Trochanter for Gluteal Muscle Contracture of the Hip,” Arthroscopy Techniques 14, no. 7 (2025): 103594. 40822185 10.1016/j.eats.2025.103594 PMC12350695

[15] J. H. Cai , L. F. Gan , H. L. Zheng , et al., “Iliac Hyperdense Line: A New Radiographic Sign of Gluteal Muscle Contracture,” Pediatric Radiology 35, no. 10 (2005): 995–997. 15965678 10.1007/s00247-005-1519-2

[16] B. Ye , P. Zhou , Y. Xia , et al., “New Minimally Invasive Option for the Treatment of Gluteal Muscle Contracture,” Orthopedics 35, no. 12 (2012): e1692–e1698. 23218623 10.3928/01477447-20121120-11

[17] J. B. Huang , H. Ge , Y. L. Zhang , et al., “The Role of Arthroscopic Release of Gluteal Muscle Contracture in Improving Patellofemoral Instability,” Journal of Orthopaedic Surgery and Research 14, no. 1 (2019): 159. 31138264 10.1186/s13018-019-1187-9 PMC6537390

[18] K. Chen , P. Gao , X. Fang , et al., “Clinical Efficacy of Modified Arthroscopic Revision Release of Gluteal Muscle Contracture With Residual Symptoms After Open Surgery,” Sichuan Da Xue Xue Bao. Yi Xue Ban = Journal of Sichuan University. Medical Science Edition 55, no. 2 (2024): 297–302. 38645866 10.12182/20240360107 PMC11026877

[19] C. G. Zhao , X. J. He , B. Lu , et al., “Classification of Gluteal Muscle Contracture in Children and Outcome of Different Treatments,” BMC Musculoskeletal Disorders 10 (2009): 34. 19351391 10.1186/1471-2474-10-34 PMC2671480

[20] D. Fu , S. Yang , B. Xiao , et al., “Comparison of Endoscopic Surgery and Open Surgery for Gluteal Muscle Contracture,” Journal of Pediatric Orthopaedics 31, no. 5 (2011): e38–e43. 21654446 10.1097/BPO.0b013e31821f509c

[21] Y. Yang , Z. Peng , L. Shi , et al., “Minimally Invasive Treatment of Grade I and II Gluteal Muscle Contracture Using a Self‐Made Special Cutter Combined With a Specialized Compression Hemostasis Device,” BMC Musculoskeletal Disorders 25, no. 1 (2024): 1024. 39702133 10.1186/s12891-024-08150-1 PMC11660755

[22] J. Hu , J. Zhu , Z. Zhou , et al., “Clinical Outcomes of Arthroscopic Surgery in Patients With Gluteal Muscle Contracture: Single‐Institution Results From a High‐Volume Cohort,” Medical Science Monitor: International Medical Journal of Experimental and Clinical Research 30 (2024): e942945. 38442083 10.12659/MSM.942945 PMC10924427

[23] D. Jia , Q. Ran , F. Sun , et al., “Effectiveness Analysis of Arthroscopic Outside‐In Release for Gluteal Muscle Contracture in Supine Position,” Zhongguo Xiu Fu Chong Jian Wai Ke Za Zhi = Zhongguo Xiufu Chongjian Waike Zazhi = Chinese Journal of Reparative and Reconstructive Surgery 39, no. 7 (2025): 848–854. 40659588 10.7507/1002-1892.202504110 PMC12279915

[24] Y. D. Wu , K. K. Yu , M. Y. An , et al., “Clinical Efficacy of the Treatment of Bilateral Gluteal Muscle Contracture by Inside‐Out Iliotibial Band Release Under Arthroscopy in the Supine Position,” Zhonghua Yi Xue Za Zhi 103, no. 21 (2023): 1611–1616. 37248060 10.3760/cma.j.cn112137-20221212-02633

[25] J. Xu , X. Geng , H. Muhammad , et al., “Comparison of the Incisions for the Open Surgical Treatment of Gluteal Muscle Contracture,” Journal of Pediatric Orthopaedics. Part B 23, no. 5 (2014): 435–440. 24887050 10.1097/BPB.0000000000000067

[26] Z. Dai , Z. Chen , Y. Liao , Z. Tang , and J. Cui , “Comparison of Arthroscopic Versus Open Surgery on External Snapping Hip Caused by Gluteal Muscle Contracture,” Hip International 28, no. 2 (2018): 173–177. 29890911 10.1177/1120700017754013

[27] X. Jiang , H. Zhang , Y. Ren , et al., “The Pattern of Collagen Production May Contribute to the Gluteal Muscle Contracture Pathogenic Process,” Journal of Orthopaedic Surgery and Research 18, no. 1 (2023): 579. 37550712 10.1186/s13018-023-04069-w PMC10408206

